# Antibiotic Use and Antimicrobial Resistance: A Global Public Health Crisis

**DOI:** 10.3390/antibiotics13090900

**Published:** 2024-09-21

**Authors:** Ana Estany-Gestal, Angel Salgado-Barreira, Juan Manuel Vazquez-Lago

**Affiliations:** 1 Health Research Institute of Santiago de Compostela (IDIS), 15706 Santiago de Compostela, Spain; 2 Department of Preventive Medicine and Public Health Service, Faculty of Pharmacy, University of Santiago de Compostela, Campus Vida s/n, 15705 Santiago de Compostela, Spain; 3 Department of Preventive Medicine and Public Health Service, University Hospital of Santiago de Compostela, Rua da Choupana s/n, 15705 Santiago de Compostela, Spain

The discovery of antibiotics revolutionized modern medicine, effectively treating bacterial infections that were once fatal. However, the widespread misuse and overuse of these drugs have led to the emergence and spread of resistant microorganisms, compromising the efficacy of current treatments [1]. The World Health Organization (WHO) has identified antimicrobial resistance as one of the top ten global health threats [2].

The indiscriminate use of antibiotics in human medicine, veterinary practices, and agriculture is a key driver of antimicrobial resistance (AMR) [3]. Antibiotics are dispensed without a prescription in many countries, facilitating unnecessary use [4]. Additionally, in the agricultural sector, the prophylactic and growth-promoting use of antibiotics in animals is widespread, significantly contributing to the spread of resistance [5]. The impact of inappropriate antibiotic use is further exacerbated by the lack of education and awareness among healthcare professionals and the general public [6,7]. A recent study found that many patients still mistakenly believe that antibiotics are effective against viral infections, such as the common cold [8]. This misunderstanding drives unnecessary demand for these drugs, pressuring healthcare providers to prescribe them even when they are not needed.

Consequently, AMR has a devastating global impact on developed and developing countries. It is estimated that antimicrobial-resistant infections cause approximately 1.27 million deaths annually [9]. Moreover, AMR prolongs the duration of illnesses, increases mortality, and imposes a significant economic burden due to the additional costs associated with prolonged treatment and hospitalization [10]. In developing countries, the impact of AMR is particularly severe due to limited access to second-line drugs, accurate diagnostics, and robust healthcare systems. Based on this premise, the Bellagio Group for Accelerating AMR Action met in April 2024 to develop the ambitious but achievable 1-10-100 unifying goals to galvanize global policy change and investments for antimicrobial resistance mitigation [11].

Addressing AMR requires a multifaceted approach that includes regulating antibiotic use, investing in R&D for new drugs, and implementing global educational programs [12]. Policies promoting the rational use of antibiotics are essential to limit inappropriate prescriptions and reduce unnecessary demand [13]. At the global level, initiatives such as the WHO Global Action Plan on Antimicrobial Resistance aim to strengthen surveillance and research, reduce infection incidence, and optimize antimicrobials in human, animal, and environmental health [14]. However, effectively implementing these strategies requires international collaboration and a firm commitment from all sectors involved.

This Special Issue presents a compendium of multidisciplinary research on the use of antibiotics, the resistance they generate, and the impact this has on a global level. The collected works serve as a comprehensive resource for scholars engaged in this field, and the Guests Editors are grateful for the interest and contributions received. 
